# Supplementary material for: Preoperative Nutrition Intervention in Patients Undergoing Resection for Upper Gastrointestinal Cancer: Results from the Multi-Centre NOURISH Point Prevalence Study
Source: Nutrients. 2021 Sep 15;13(9):3205. doi: 10.3390/nu13093205 (PMC8467838; doi:10.3390/nu13093205)
Supplement: Supplementary file 1 [file nutrients-13-03205-s001.zip › nutrients-1371132-supplementary file.pdf]

The NOURISH Point Prevalence Study Data Collection Tool V3 13.5.19

Record ID

____
(Use Study Number provided on master re-identifier list)

PATIENT REGISTRATION AND CONSENT

Consent and face to face data collection must occur within 7 days of surgery (surgery is Day 0)

Is the patient eligible to participate in the study?

◯ Yes
◯ No
(Review the eligibility criteria. If no, then the patient is excluded and no further data can be collected)

Reason for ineligibility

◯ Aged < 18 years.
◯ Underwent surgery for palliative intent.
◯ Unable to provide consent or participate in assessment, including due to being too unwell.
◯ Taking intravenous (IV) opioids including patient controlled analgesia (PCA) at the time of recruitment.
◯ Unaware of their diagnosis of malignancy.
(Record why the patient was ineligible to participate)

Date of approaching patient to participate in the study

____
(Enter as DDMMYYYY)

Was the patient provided with the Patient information Sheet?

◯ Yes
◯ No
(If no, do not ask for verbal consent until the patient receives and has read it)

Was verbal consent given?

◯ Yes
◯ No
(if no, then no further data can be collected)

Primary Language Spoken

____

Was an interpreter used?

◯ Yes
◯ No

Source of interpreter

◯ Family or friend
◯ Professional

PATIENT INFORMATION<br>Collect from medical record	
Sex	◯ Male<br>◯ Female
Age	______________________________<br>(age on date of surgery)
Postcode of usual residence	______________________________
Date of this admission	______________________________<br>(Enter as DDMMYYYY)
Date of Surgery	______________________________<br>(Enter as DDMMYYYY)
Surgical Procedure	◯ Oesophagectomy<br>◯ Partial gastrectomy<br>◯ Subtotal gastrectomy<br>◯ Total gastrectomy<br>◯ Pancreaticoduodenectomy (whipple's)<br>◯ Partial pancreatectomy<br>◯ Distal pancreatectomy<br>◯ Total pancreatectomy<br>◯ Other<br>(Record from surgical report. Can record multiple if applicable)
Surgical Technique	◯ Open or converted to open<br>◯ Laparoscopic<br>◯ Minimally invasive<br>◯ Hybrid of laparoscopic and minimally invasive
Tumour Location	◯ Gastric<br>◯ Oesophageal<br>◯ Pancreatic<br>◯ Ampullary<br>◯ Duodenal<br>◯ Small intestinal<br>◯ Other
Tumour Type	◯ Adenocarcinoma<br>◯ Squamous cell carcinoma<br>◯ Gastrointestinal stromal tumour (GIST)<br>◯ Neuroendocrine tumour (NET)<br>◯ Other<br>(Record from histopathology report)
Intraoperative Tumour Stage	◯ T1<br>◯ T2<br>◯ T3<br>◯ T4<br>◯ Unsure<br>(Record from histopathology report)

Intraoperative Tumour Nodes positive

____
(Record from histopathology report. Whole number from 0. if unsure, leave BLANK)

Intraoperative Tumour Metastases

____
(Record from histopathology report. Whole number from 0. if unsure, leave BLANK)

Month and Year of Initial Diagnosis (if known)

____
(Enter as MMYYYY. cross check with patient if information unclear from medical records)

Did the patient receive any neoadjuvant treatment?

◯ Yes
◯ No
◯ Unsure
(cross check with patient if information unclear from medical records)

Did the patient complete their neoadjuvant treatment?

◯ Yes
◯ No
◯ Unsure
◯ Did not receive neoadjuvant
(cross check with patient if information unclear from medical records)

Type of neoadjuvant received

◯ Chemotherapy
◯ Radiotherapy
◯ Chemotherapy and Radiotherapy
◯ Unsure
(cross check with patient if information unclear from medical records)

Usual social situation

◯ Lives alone
◯ Lives with family or carer
◯ Lives in shared accommodation
◯ Lives in residential care
(cross check with patient if information unclear from medical records)

Identifies as Aboriginal or Torres Straight Islander

◯ Yes
◯ No
◯ Unsure
(cross check with patient if information unclear from medical records)

NUTRITIONAL ASSESSMENT
Must be collected face to face with the patient; within the 7 day post surgery period.
Remember, can use already routinely collected information to reduce patient burden if patient has consented to this. See Consent procedures.

Day post surgery of nutritional assessment (Day 0 is day of surgery)

____

Height (cm, nearest 0.1 cm)	__________________________ (Dietitian to clarify by asking patient or by measuring usual a height stadiometer. If patient unable, recorded by dietitian from medical record if available.)
Current Weight (kg, nearest 0.5 kg)	__________________________ (Dietitian to weight patient, or obtain from the medical history if weighed within first 3 days of admission. If unable to weigh patient, dietitian to ask patient what their weight was the week before surgery, and check the medical history. Ensure that if patient appears oedematous, then dry weight is recorded by estimation and by clarifying with the patient what their weight was the week before surgery.)
Body Mass Index (BMI)	__________________________ (calculate as per formula)
Is the BMI criteria met for ICD-10AM diagnosis of malnutrition?	◯ YES BMI < 18.5kg/m2<br>◯ NO<br>(Tick YES if BMI < 18.5kg/m2 as per ICD-10 AM criteria)
Has the patient lost any weight recently?	◯ No<br>◯ Yes and amount known<br>◯ Yes and amount unknown<br>◯ Unsure<br>(Dietitian to ask patient. If patient is unsure of any weight loss, or amount of weight loss, dietitian to check the medical history. If not information available from the medical record, tick UNSURE)
How much weight has the patient lost? (kg, nearest 0.5kg)	__________________________ (If unsure, leave blank. Check medical records if patient unsure)
Is the weight loss intentional or unintentional?	◯ Intentional<br>◯ Unintentional<br>◯ Unsure<br>(Dietitian to ask patient.)
Weight 2 weeks ago (kg, nearest 0.5 kg)	__________________________ (Check medical records if patient unsure. If no information, leave blank)
Weight 1 month ago (kg, nearest 0.5kg)	__________________________ (Check medical records if patient unsure. If no information, leave blank)

Weight 3 months ago (kg, nearest 0.5kg)

____
(Check medical records if patient unsure. If no information, leave blank)

Weight 6 months ago (kg, nearest 0.5kg)

____
(Check medical records if patient unsure. If no information, leave blank)

Weight >6 months ago (kg, nearest 0.5kg)

____
(Check medical records if patient unsure. If no information, leave blank)

Percentage Weight loss in 1 month (nearest 1%)

____
(If unsure/no information, leave blank)

Percentage weight loss in 3 months (nearest 1%)

____
(If unsure/no information, leave blank)

Percentage weight loss in 6 months (nearest 1%)

____
(If unsure/no information, leave blank)

Percentage weight loss in > 6 months (nearest 1%)

____
(If unsure/no information, leave blank)

Is the % weight loss criteria met for malnutrition as per ICD10-AM criteria?

◯ Yes ≥ 5% LOW reported (mild-moderate)
◯ Yes > 10% LOW reported (severe)
◯ No < 5% LOW reported or intentional LOW
◯ Unsure

Did the patient have a reduced food intake before their surgical admission?

◯ Yes
◯ No
◯ Unsure
(Dietitian to ask patient.)

To what degree had their food intake been reduced?

◯ >75% of their usual intake
◯ ≤ 75% of their usual intake
◯ ≤ 50% of their usual intake
◯ ≤ 25% of their usual intake
(Dietitian to ask patient.)

How long had their food intake been reduced?

◯ < 1 week
◯ 1-2 weeks
◯ 2-4 weeks
◯ ≥1 month
(Dietitian to ask patient.)

has the patient reported any gastrointestinal symptoms persisting > 2 weeks prior to surgery that have been impacting their ability to eat?	☐ Poor appetite<br>☐ Nausea<br>☐ Vomiting<br>☐ Diarrhoea<br>☐ Constipation<br>☐ Pain when eating<br>☐ Taste changes<br>☐ Dry mouth<br>☐ Problems swallowing<br>☐ Early satiety<br>(Dietitian to ask patient. Can record multiple symptoms)
Activity level in the past month before surgery	◯ Normal activities, with no limitations<br>◯ Not normal self, but able to be up and about with fairly normal activities (>75% of normal)<br>◯ Not feeling up to most things, but in bed or chair less than half of the day (50-75% normal)<br>◯ Spending most of the day in the bed or chair, but do a little amount of activity (25-50%)<br>◯ Pretty much bed ridden (< 25%)<br>(Dietitian to ask patient.)

PHYSICAL ASSESSMENT

Consent for physical assessment	◯ Yes<br>◯ No<br>(If no, then no physical assessment can be conducted)

Muscle Loss Assessment

Aim to examine each individual site/area if possible. If not possible, aim to examine ≥4 muscle sites

Leave sites blank if not assessed

	NO DEFICIT	MILD-MODERATE	SEVERE
Temples	◯	◯	◯
Clavicles	◯	◯	◯
Shoulders	◯	◯	◯
Interosseous muscle	◯	◯	◯
Scapula	◯	◯	◯
Thigh	◯	◯	◯
Calf	◯	◯	◯

Overall Muscle Loss Assessment	◯ No Deficit<br>◯ Mild-Moderate Deficit<br>◯ Severe Deficit

Fat Loss Assessment
Aim to examine each individual site/area if possible. If not possible, aim to examine ≥2 fat sites
Leave sites blank if not assessed

	NO DEFICIT	MILD-MODERATE	SEVERE
Orbital	◯	◯	◯
Triceps	◯	◯	◯
Fat Overlying Ribs	◯	◯	◯

Overall fat loss assessment
- ◯ No deficit
- ◯ Mild-Moderate deficit
- ◯ Severe deficit

Fluid Accumulation Assessment
Aim to examine each individual site/area if possible. If not possible, aim to examine ≥2 fluid accumulation sites
Leave sites blank if not assessed

	NO FLUID	MILD-MODERATE	SEVERE
ankle or sacral odema	◯	◯	◯
ascites	◯	◯	◯

Overall fluid accumulation assessment
- ◯ No fluid accumulation
- ◯ Mild-Moderate fluid accumulation
- ◯ Severe fluid accumulation

MALNUTRITION DIAGNOSIS

Subjective Global Assessment Rating
- ◯ A No Malnutrition
- ◯ B Mild/Moderate or Suspected Malnutrition
- ◯ C Severe Malnutrition

Is the patient malnourished according to ICD-10AM criteria?
- ◯ NO MALNUTRITION. BMI > 18.5 kg/m2 and unintentional loss of weight < 5%, with no evidence of suboptimal intake or fat/muscle wasting.
- ◯ YES MILD MODERATE. BMI < 18.5 kg/m2 or unintentional loss of weight (5-9%) with evidence of suboptimal intake resulting in moderate loss of subcutaneous fat and/or moderate muscle wasting.
- ◯ YES SEVERE. BMI < 18.5 kg/m2 or unintentional loss of weight (> 10%) with evidence of suboptimal intake resulting in severe loss of subcutaneous fat and/or severe muscle wasting

(IF YES PATIENT REQUIRES DIETETIC INPUT AS PER SITE SPECIFIC REFERRAL PROCEDURES)

HAND GRIP STRENGTH ASSESSMENT

Consent for hand grip strength assessment

◯ Yes
◯ No
◯ Not available at this site
(if no, then no hand grip data can be collected)

Hand grip strength score average left (kg, to 2 decimal places)

____

Hand grip strength score average right (kg, to 2 decimal places)

____

Does the patient have Low muscle strength?(using highest average value of left and right)

◯ YES < 20 kg women or < 30kg for men
◯ NO
◯ NA

PRE OPERATIVE DIETETICS INTERVENTION

Collect from patient

Has the patient seen a dietitian at all pre-surgery (since diagnosis)?

◯ Yes
◯ No
◯ Unsure
(Dietitian to ask patient. If patient is unsure, check medical record. If no information available then tick UNSURE)

What was the location of this dietitian service?

◯ In chemotherapy
◯ In radiotherapy
◯ During an inpatient admission
◯ Onsite UGI outpatient clinic
◯ Onsite general nutrition outpatient clinic
◯ Community health service
◯ Private dietitian
◯ Other
(Dietitian to ask patient. If patient is unsure, check medical record. If no information available then tick UNSURE)

If other, please specify

____

if yes, how many appointments did the patient have?

◯ 1 appointment
◯ 2 appointments
◯ 3-4 appointments
◯ >4 appointments
◯ Unsure
(Dietitian to ask patient. If patient is unsure, check medical record. If no information available then tick UNSURE)

If yes, when was the last appointment?

◯ 1-2 weeks before surgery
◯ 2-4 weeks before surgery
◯ > 1 month before surgery
◯ > 3 months before surgery
(Dietitian to ask patient. If patient is unsure, check medical record. If no information available then tick UNSURE)

Has the patient received any nutritional advice from a surgeon, physician or other health care professional prior to surgery?	◯ Yes<br>◯ No<br>◯ Unsure<br>(Dietitian to ask patient. If patient is unsure, check medical record. If no information available then tick UNSURE)
What type of advice was provided?	◯ Advice so they can gain weight<br>◯ Advice so they can lose weight<br>◯ High protein<br>◯ Nutritional supplement drinks<br>(Dietitian to ask patient. If patient is unsure, check medical record. If no information available then tick UNSURE)
Was the patient taking any nutritional supplement drinks prior to surgery?	◯ Yes<br>◯ No<br>◯ Unsure<br>(Dietitian to ask patient. If patient is unsure, check medical record. If no information available then tick UNSURE)
If yes, what type?	☐ High protein/calorie drinks<br>☐ Immunonutrition drinks<br>☐ Carbohydrate loading drinks<br>☐ UNSURE<br>(Dietitian to ask patient. Can record multiple options)
If yes to high protein drinks, how long was the patient taking them for?	◯ 5 days before surgery<br>◯ 1 week before surgery<br>◯ >2 weeks before surgery<br>◯ > 1 month before surgery<br>◯ > 3 months before surgery<br>(Dietitian to ask patient. If patient is unsure, check medical record. If no information available then tick UNSURE)
If yes to immunonutrition drinks, how long was the patient taking them for?	◯ 5 days before surgery<br>◯ 1 week before surgery<br>◯ >2 weeks before surgery<br>◯ > 1 month before surgery<br>◯ > 3 months before surgery<br>(Dietitian to ask patient. If patient is unsure, check medical record. If no information available then tick UNSURE)

INPATIENT NUTRITIONAL OUTCOMES

Collect from medical record

Consent for collection of remainder of information from the medical history during the patient's admission?	◯ Yes<br>◯ No<br>(if no, then no further data can be collected)
Was a feeding tube inserted intra-operatively?	◯ Yes<br>◯ No<br>◯ No- already present pre surery

What was the date of insertion of this feeding tube if inserted pre-surgery?

____
(Enter as DDMMYYYY)

If yes, what type of feeding tube is it?

- ◯ NASOJEJUNAL
- ◯ NASOGASTRIC
- ◯ JEJUNOSTOMY
- ◯ GASTROSTOMY
- ◯ TRANSGASTRIC JEJUNOSTOMY

What day of admission was the first dietetic contact?

____
(Whole number from 0. If no dietetics care received, leave blank)

Estimated energy requirements per day (kj)

____
(whole number. If calculated a range, record the average of that range)

Estimated protein requirements per day (g)

____
(whole number. If calculated a range, record the average of that range)

Diet Codes First 10 Days of Admission

Collect from medical record

	Nil by mouth	Clear Fluids	Free Fluids	Pureed or Minced	Light ward diet	Soft ward diet	Full ward diet
Day 0	◯	◯	◯	◯	◯	◯	◯
Day 1	◯	◯	◯	◯	◯	◯	◯
Day 2	◯	◯	◯	◯	◯	◯	◯
Day 3	◯	◯	◯	◯	◯	◯	◯
Day 4	◯	◯	◯	◯	◯	◯	◯
Day 5	◯	◯	◯	◯	◯	◯	◯
Day 6	◯	◯	◯	◯	◯	◯	◯
Day 7	◯	◯	◯	◯	◯	◯	◯
Day 8	◯	◯	◯	◯	◯	◯	◯
Day 9	◯	◯	◯	◯	◯	◯	◯
Day 10	◯	◯	◯	◯	◯	◯	◯

Dietetics Intervention first 10 days of admission
Collect during routine clinical care. If you haven't seen the patient on a particular day, record what intervention was prescribed at the last dietitian review

	No nutrition intervention prescribed	Oral nutrition supplements	Enteral nutrition support	Parenteral nutrition support	HEHP diet	Dietary Education
Day 0	☐	☐	☐	☐	☐	☐
Day 1	☐	☐	☐	☐	☐	☐
Day 2	☐	☐	☐	☐	☐	☐
Day 3	☐	☐	☐	☐	☐	☐
Day 4	☐	☐	☐	☐	☐	☐
Day 5	☐	☐	☐	☐	☐	☐
Day 6	☐	☐	☐	☐	☐	☐
Day 7	☐	☐	☐	☐	☐	☐
Day 8	☐	☐	☐	☐	☐	☐
Day 9	☐	☐	☐	☐	☐	☐
Day 10	☐	☐	☐	☐	☐	☐

Percentage Energy requirements met for the first 10 days of admission
Collect during routine clinical care. If you haven't seen the patient on a particular day, record an average of intake since the last review

	< 20%	20-40%	40-60%	60-80%	80-100%
Day 0	○	○	○	○	○
Day 1	○	○	○	○	○
Day 2	○	○	○	○	○
Day 3	○	○	○	○	○
Day 4	○	○	○	○	○
Day 5	○	○	○	○	○
Day 6	○	○	○	○	○
Day 7	○	○	○	○	○
Day 8	○	○	○	○	○
Day 9	○	○	○	○	○
Day 10	○	○	○	○	○

Percentage protein requirements met for the first 10 days of admission
Collect during routine clinical care. If you haven't seen the patient on a particular day, record an average of intake since the last review

	< 20%	20-40%	40-60%	60-80%	80-100%
Day 1	◯	◯	◯	◯	◯
Day 2	◯	◯	◯	◯	◯
Day 3	◯	◯	◯	◯	◯
Day 4	◯	◯	◯	◯	◯
Day 5	◯	◯	◯	◯	◯
Day 6	◯	◯	◯	◯	◯
Day 7	◯	◯	◯	◯	◯
Day 8	◯	◯	◯	◯	◯
Day 9	◯	◯	◯	◯	◯
Day 10	◯	◯	◯	◯	◯

Weight within 2 days of discharge (kg, nearest 0.5kg)

____
(If not completed, leave blank)

DISCHARGE DIETETICS OUTCOMES
Collect from medical record

Total number of contacts with dietitian during inpatient admission

____

Diet Code for Discharge

- ◯ Nil by mouth
- ◯ Clear Fluids
- ◯ Free Fluids
- ◯ Pureed or Minced
- ◯ Light ward diet
- ◯ Soft ward diet
- ◯ Full ward diet
- ◯ Diet code not known

Oral Nutrition Supplements prescribed on discharge?

- ◯ Yes
- ◯ No
- ◯ Unsure

If yes, what % of energy and protein requirements does this aim to meet per day?

- ◯ Not prescribed
- ◯ Unsure
- ◯ < 20%
- ◯ 20-40%
- ◯ 40-60%
- ◯ 60-80%
- ◯ 80-100%

Enteral Nutrition prescribed on discharge?

- ◯ Yes
- ◯ No
- ◯ Unsure

If yes, what % of energy and protein requirements does this aim to meet per day?	◯ Not prescribed<br>◯ Unsure<br>◯ < 20%<br>◯ 20-40%<br>◯ 40-60%<br>◯ 60-80%<br>◯ 80-100%
Dietetics follow up arranged post discharge?	◯ No follow up arranged<br>◯ Follow up arranged, timeframe not specified<br>◯ Within 2 weeks<br>◯ Within 2-4 weeks<br>◯ Within 4-8 weeks<br>◯ Longer than 8 weeks
Dietetics Follow up location	◯ No follow up arranged<br>◯ On site specialist UGI clinic<br>◯ On site general nutrition clinic<br>◯ CHS dietitian<br>◯ Private dietitian<br>◯ Location not specified<br>◯ Other
if other, specify	__________________________

SURGICAL OUTCOMES
Collect from medical record

Inpatient admission discharge date	__________________________<br>(Enter as DDMMYYYY)
Discharge Destination	◯ Home<br>◯ Aged care facility<br>◯ Inpatient rehabilitation facility<br>◯ Respite care<br>◯ Death<br>◯ Unknown
Did the patient develop any of the following complications during their surgical admission?	◯ Surgical site infection or wound infection<br>◯ Sepsis<br>◯ Anastomotic leak<br>◯ Fistula<br>◯ Pneumonia<br>◯ Respiratory Tract Infection<br>◯ Pressure Injury<br>◯ Wound dehiscence<br>◯ Return to theatre<br>◯ Abdominal collection<br>◯ Ileus<br>(can record multiple complications)
FREE TEXT FOR ANY COMMENTS RELATING TO THIS PATIENT'S DATA COLLECTION	__________________________
