# Supplementary material for: Preoperative Nutrition Intervention in Patients Undergoing Resection for Upper Gastrointestinal Cancer: Results from the Multi-Centre NOURISH Point Prevalence Study
Source: Nutrients. 2021 Sep 15;13(9):3205. doi: 10.3390/nu13093205 (PMC8467838; doi:10.3390/nu13093205)
Supplement: Supplementary file 1 [file nutrients-13-03205-s001.zip › nutrients-1371132-supplementary.pdf]

Supplementary Table S1. Multivariate analysis of preoperative dietetics intervention (≥3 appointments) with surgical outcomes in malnourished patients (n=84).

Variable	LOS<br>Coefficient (95% CI)	P value	Complications<br>OR (95% CI)	P value
**≥3 dietetics appointments**	1.5 (-6.4, 9.4)	0.703	0.3 (0.1, 0.9)	**0.040**
**Age ≥65**	1.0 (-6.8, 8.8)	0.802	2.7 (0.8, 9.1)	0.115
**Tumour Stage**				
T1	-12.2 (-31.1, 6.7)	0.201	0.3 (0.1, 4.8)	0.350
T2	-3.6 (-22.1, 14.9)	0.700	0.7 (0.1, 14.0)	0.835
T3	-9.6 (-27.5, 8.4)	0.291	0.6(0.1, 10.7)	0.744
T4	-9.3 (-30.1, 11.4)	0.372	0.4 (0.1, 12.4)	0.626
**Surgery Type**				
Gastrectomy	-4.4 (-15.3, 6.5)	0.426	0.1 (0.1, 0.6)	**0.016**
Pancreatectomy	-0.6 (-10.0, 8.7)	0.892	0.3 (0.1, 1.1)	0.073
**≥5% LOW in 6 months**	3.1 (-6.2, 12.4)	0.506	0.5 (0.1, 2.3)	0.399

OR=Odds Ratio, LOW=Loss of weight.

Supplementary Table S2. Multivariate analysis of preoperative nutrition support (HEHP supplements > 2 weeks) with surgical outcomes in malnourished patients (n=84).

Variable	LOS<br>Coefficient (95% CI)	P value	Complications<br>OR (95% CI)	P value
**HEHP > 2 weeks received**	-7.3 (-14.3, -0.3)	**0.041**	0.5 (0.2, 1.5)	0.204
**Age ≥65**	2.8 (-4.8, 10.4)	0.462	3.1 (0.9, 10.5)	0.073
**Tumour Stage**				
T1	-10.6 (-26.5, 5.3)	0.187	0.4 (0.1, 4.7)	0.473
T2	-1.5 (-17.0, 14.0)	0.850	1.5 (0.1, 15.7)	0.754
T3	-7.6 (-22.4, 7.2)	0.306	1.2 (0.1, 11.0)	0.895
T4	-6.1 (-23.2, 11.0)	0.480	0.6 (0.1, 9.2)	0.706
**Surgery Type**				
Gastrectomy	-7.1 (-16.6, 2.4)	0.140	0.1 (0.1, 0.7)	**0.016**
Pancreatectomy	-4.5 (-12.7, 3.8)	0.286	0.4 (0.1, 1.3)	0.129
**≥5% LOW in 6 months**	1.1 (-7.8, 10.0)	0.804		

HEHP=High Energy High Protein, OR=Odds Ratio, LOW=Loss of weight.
